# Plasma acylcarnitine profiling indicates increased fatty acid oxidation relative to tricarboxylic acid cycle capacity in young, healthy low birth weight men

**DOI:** 10.14814/phy2.12977

**Published:** 2016-09-30

**Authors:** Amalie Ribel‐Madsen, Rasmus Ribel‐Madsen, Charlotte Brøns, Christopher B. Newgard, Allan A. Vaag, Lars I. Hellgren

**Affiliations:** ^1^ Department of Biotechnology and BiomedicineTechnical University of DenmarkKongens LyngbyDenmark; ^2^ Department of Endocrinology, Diabetes and MetabolismRigshospitalet, Copenhagen University HospitalCopenhagenDenmark; ^3^ Danish Diabetes AcademyOdenseDenmark; ^4^ Sarah W. Stedman Nutrition and MetabolismCenter and Duke Molecular Physiology InstituteDuke UniversityDurhamNCUSA

**Keywords:** Acylcarnitines, high‐fat overfeeding, low birth weight, type 2 diabetes

## Abstract

We hypothesized that an increased, incomplete fatty acid beta‐oxidation in mitochondria could be part of the metabolic events leading to insulin resistance and thereby an increased type 2 diabetes risk in low birth weight (LBW) compared with normal birth weight (NBW) individuals. Therefore, we measured fasting plasma levels of 45 acylcarnitine species in 18 LBW and 25 NBW men after an isocaloric control diet and a 5‐day high‐fat, high‐calorie diet. We demonstrated that LBW men had higher C2 and C4‐OH levels after the control diet compared with NBW men, indicating an increased fatty acid beta‐oxidation relative to the tricarboxylic acid cycle flux. Also, they had higher C6‐DC, C10‐OH/C8‐DC, and total hydroxyl‐/dicarboxyl‐acylcarnitine levels, which may suggest an increased fatty acid omega‐oxidation in the liver. Furthermore, LBW and NBW men decreased several acylcarnitine levels in response to overfeeding, which is likely a result of an upregulation of fatty acid oxidation due to the dietary challenge. Moreover, C10‐OH/C8‐DC and total hydroxyl‐/dicarboxyl‐acylcarnitine levels tended to be negatively associated with the serum insulin level, and the total hydroxyl‐/dicarboxyl‐acylcarnitine level additionally tended to be negatively associated with the hepatic insulin resistance index. This indicates that an increased fatty acid omega‐oxidation could be a compensatory mechanism to prevent an accumulation of lipid species that impair insulin signaling.

## Introduction

Low birth weight (LBW) individuals have an increased risk of developing obesity, cardiovascular disease, and type 2 diabetes, compared with normal birth weight (NBW) individuals, when exposed to an affluent life style such as overfeeding (Ravelli et al. 1976; Hales et al. 1991; Barker et al. 1993; Harder et al. 2007). In a short‐term high‐fat overfeeding study in young, healthy LBW and NBW men, we have shown that LBW men display a number of metabolic abnormalities relevant to the pathophysiology of type 2 diabetes, including a decreased hepatic insulin sensitivity (Brons et al. 2008) prior to, and development of a decreased peripheral insulin sensitivity in response to a high‐fat, high‐calorie diet intervention (Brons et al. 2012). Furthermore, we have shown that LBW men exhibit an increased fatty acid oxidation rate, but an unaltered total energy expenditure, during night time compared with NBW men (Brons et al. 2013). However, the extent to which the disproportionately increased fatty acid oxidation rate could contribute to the decreased insulin sensitivity in LBW men as a result of a differential and potentially incomplete fatty acid oxidation remains to be studied.

High‐fat overfeeding and an increased lipid exposure to skeletal muscle has been shown to lead to an increased expression of genes in the fatty acid beta‐oxidation pathway, including the gene encoding carnitine palmitoyl‐transferase I (CPT‐I) that catalyzes the condensation of activated long‐chain fatty acids (acyl‐CoAs) to carnitine to form acylcarnitines and thereby regulates the entry of these acyl‐CoAs into the mitochondrial matrix (Koves et al. 2005; Muoio and Newgard 2006; Turner et al. 2007). Also, in the state of high‐fat overfeeding, an increased beta‐oxidation has been suggested to not necessarily be matched by increased tricarboxylic acid (TCA) cycle and electron transport chain fluxes, which results in an incomplete fatty acid oxidation (Koves et al. 2005, 2008; Muoio and Newgard 2006, 2008b). This leads to an accumulation of acylcarnitines and reactive oxygen species that may contribute to metabolic stress and thereby ultimately impair insulin signaling (Bloch‐Damti and Bashan 2005; Adams et al. 2009; Rutkowsky et al. 2014; Aguer et al. 2015). In addition to an incomplete beta‐oxidation, increased intracellular concentrations of long‐chain acyl‐CoAs may lead to an increased lipogenesis, hereunder the synthesis of lipid species that impair insulin signaling (Muoio and Newgard 2008b). This metabolic fate of long‐chain acyl‐CoAs has been especially described in the context of high‐fat diet‐induced hepatic insulin resistance. Actually, high‐fat overfeeding has been proposed to lead to malonyl‐CoA induced inhibition of CPT‐I activity in the liver, and a following diversion of long‐chain acyl‐CoAs away from beta‐oxidation and toward other metabolic fates in the cytosol, including lipogenesis (Muoio and Newgard 2008a,b). However, studies also point to that an increased lipid exposure to the liver leads to a simultaneously increased beta‐oxidation and incorporation of long‐chain acyl‐CoAs into lipids (Ciapaite et al. 2011).

Incomplete fatty acid beta‐oxidation downstream of CPT‐I is reflected by elevated plasma acylcarnitine levels (Koves et al. 2008), as acyl‐CoAs in the mitochondrial matrix can be converted into acylcarnitines that subsequently are transported through the mitochondrial membranes and thereafter from the cytosol to the blood (Koves et al. 2008; Millington and Stevens 2011; Violante et al. 2013). Actually, higher plasma acylcarnitine levels have been found in adults with prediabetes and type 2 diabetes (Adams et al. 2009; Mihalik et al. 2010; Ha et al. 2012; Mai et al. 2013). Also, defects in specific steps of beta‐oxidation can be revealed by altered acylcarnitine levels, as made use of in the diagnosis of inborn errors in fatty acid metabolism (Millington et al. 1990; Chace et al. 2001, 2003). Moreover, the involvement of other metabolic pathways upstream of beta‐oxidation may be reflected in the composition and concentrations of acylcarnitine species (Koves et al. 2008). Thus, an incomplete beta‐oxidation give rise to even‐chain C4–C22 acylcarnitine species, and amino acid catabolism is a source for C3, C4, and C5 species (Koves et al. 2008). These pathways are, together with glucose oxidation, in addition sources of acetylcarnitine, C2, when acetyl‐CoA is generated in excess in the mitochondrial matrix relative to the flux into the TCA cycle (Zammit 1999; Koves et al. 2008). In this situation, carnitine acetyl‐CoA transferase (CrAT) catalyzes the transfer of acetyl‐CoA to carnitine to form acetylcarnitine, which is subsequently transported to the cytosol (Zammit 1999; Muoio Deborah et al. 2012). An accumulation of acyl‐CoAs in the cytosol due to an incomplete beta‐oxidation may lead to an increased fatty acid omega‐oxidation in the endoplasmic reticulum of mainly the liver (Bjorkhem 1976; Reddy and Hashimoto 2001; Patsouris et al. 2006). This latter is expected to be reflected in higher plasma hydroxyl‐/dicarboxyl‐acylcarnitine levels (Reddy and Hashimoto 2001; Houten et al. 2012).

We hypothesized that an increased, incomplete fatty acid beta‐oxidation could contribute to the impaired insulin sensitivity in LBW individuals, reflected by elevated plasma acylcarnitine levels. Accordingly, we analyzed fasting plasma levels of 45 acylcarnitine species, including even‐chain C2–C22 species, odd‐chain C3–C7 species, and hydroxyl‐/dicarboxyl‐species, in LBW and NBW men following an isocaloric control diet and a 5‐day high‐fat, high‐calorie diet.

## Materials and Methods

### Study population

Forty‐six young (23–27 years of age), healthy men were recruited from the Danish National Birth Registry according to birth weight. All individuals were born at term (39–41 weeks of gestation) and in Copenhagen in the period 1979–1980. LBW was defined as a birth weight below the 10th percentile, as earlier studies have shown that individuals within this range have an increased risk of developing type 2 diabetes (Jaquet et al. 2000; Jensen et al. 2002), and NBW was defined as a birth weight within the 50th–90th percentile range. Among the recruited men, 20 had LBW (2717 ± 268 g) and 26 had NBW (3901 ± 207 g). Furthermore, all participants were ensured to not have a family history of diabetes in two generations, not have a body mass index (BMI) greater than 30 kg/m^2^, not perform strenuous physical activity more than 10 h per week, not take pharmaceuticals that affect metabolism, and not have an abuse of alcohol or drugs.

### Study design

#### Diet interventions

All individuals were in a randomized crossover setup standardized with respect to diet and physical activity and following given a 3‐day control diet and a 5‐day high‐fat, high‐calorie diet separated by a 6–8 weeks wash‐out period. Energy requirements of the individual subjects were calculated from a World Health Organization equation for men less than 30 years of age and a physical activity level of 1.4 corresponding to a low physical activity (WHO, 2001). The control diet was composed to reflect a habitual, weight‐maintaining diet (2819 ± 238 kcal/11,800 ± 1000 kJ) with 15% of the total energy from protein, 50% from carbohydrate, and 35% from fat, and the high‐fat, high‐calorie diet was prepared to contain 50% extra calories (4228 ± 334 kcal/17,700 ± 1,400 kJ) with 7.5% of the total energy from protein, 32.5% from carbohydrate, and 60% from fat (Table S1). Both diets were provided as five daily servings with 25% of the total energy from breakfast, 10% from morning snack, 25% from lunch, 10% from afternoon snack, and 30% from dinner, and the meals were identical from day to day. Dietary calculations were made in Dankost Pro (http://dankost.dk/english) (The National Food Agency, Copenhagen, Denmark).

#### Clinical examinations

Study activities were carried out over 3 days, with the first of these days being placed 1 or 3 days after the start of the control and high‐fat, high‐calorie diet intervention, respectively. Anthropometry was performed on the first study day. An intravenous glucose tolerance test (IVGTT) and a hyperinsulinemic‐euglycemic clamp were carried out in the morning on the third study day following an overnight fast to assess insulin secretion and sensitivity, as previously described (Brons et al. 2008, 2012). Furthermore, calorimetry was performed throughout 24 h from the first to second study day by use of a respiratory chamber and in the basal and insulin‐stimulated steady‐state periods of the clamp to evaluate substrate utilization rates and energy expenditures, as previously described (Brons et al. 2012, 2013, 2015). Blood samples were collected prior to and during the clamp.

### Laboratory measurements

#### Acylcarnitine analyses

Acylcarnitine analyses were performed on EDTA‐plasma samples collected following an overnight fast (10.00 pm–7.00 am) and immediately prior to the clamp examination. These analyses included a semi‐quantitative determination of 45 a priori selected acylcarnitine species or sets of species (ions with equal mass), noted in this text by their acyl group in accordance to its carbon chain length (e.g., C16), possible double bonds (e.g., C16:1), and possible hydroxyl‐ or a second carboxyl‐group (e.g., C16‐OH or C16‐DC, respectively) (Table 3, Table S2), and were performed by use of sample preparation procedures and flow injection‐tandem mass spectrometry (MS/MS), as previously described (An et al. 2004; Ferrara et al. 2008; Millington and Stevens 2011). In brief, plasma samples were spiked with a selection of deuterium‐labeled acylcarnitine standards, including D3‐C2, D3‐C3, D3‐C4, D9‐C5, D3‐C8, and D3‐C16 carnitines (Cambridge Isotope Laboratories, Andover, MA). Following, proteins were removed by precipitation with methanol, and the supernatants were then transferred to a 96‐well plate, evaporated to dryness under nitrogen gas, and incubated with either acidified methanol or butanol to form methyl and butyl ester derivatives of the acylcarnitines, respectively. After this, the reagents were evaporated to dryness under nitrogen gas, and the residues were redissolved in 85:15 (v/v) methanol:water. Subsequently, the samples were introduced into a Quattro Micro MS/MS system (Waters, Milford, MA) equipped with a model HTS‐PAL autosampler (Leap Technologies, Carrboro, NC) and a model 1100 HPLC solvent delivery system (Agilent Technologies, Santa Clara, CA). Mass spectra of the acylcarnitine esters were obtained by positive precursor ion scanning of m/z 99 and m/z 85 for methyl or butyl esters, respectively. Following, acylcarnitines were identified from the peaks of these derivatives and quantified from the ratio of their molecular signals to respective internal standards (Table S2). Some acylcarnitine species shared the same internal standard due to the limited number of commercially available analytical standards. Addition of more internal standards, however, does not appear to significantly improve the analytical precision (Millington and Stevens 2011). Mass spectra were analyzed by use of MassLynx 4.0 (Waters). Acylcarnitine analyses were performed in The Sarah W. Stedman Nutrition and Metabolism Center Metabolomics/Biomarker Core Laboratory, Duke University, Durham, NC. The laboratory was blinded to the birth weight of the individuals.

### Ethical approval

All study procedures were in accordance with the principles of The Declaration of Helsinki and were approved by The Regional Research Ethics Committee of Copenhagen, Denmark. Also, all participants were provided with written information on the study purpose and procedures and signed an informed consent prior to their participation.

### Statistical analyses

#### Acylcarnitine levels and their relation to physiological measures

Differences in plasma acylcarnitine levels between NBW and LBW individuals within each diet or between the control and high‐fat, high‐calorie diets within each birth weight group were assessed from Student's unpaired or paired *t*‐test (for normally distributed values), respectively, or Wilcoxon ranked‐sum or signed‐rank test (for not normally distributed values), respectively. Prior to these tests, statistical outliers (1.5 interquartile range) were removed from the dataset. Also, values below the lower limit of detection were replaced by 0.5 times this limit, which was defined as the minimum value for the actual metabolite. Following, outliers were replaced by the mean value within the group. Normal distribution of values (variables or differences between variables, respectively) was evaluated from Shapiro–Wilk test. Finally, after *P*‐values were calculated, adjustment for multiple testing was done by calculating false discovery rates, *Q*‐values, by the Benjamini and Hochberg method (Benjamini and Hochberg 1995). Data in Table 3 are presented as mean value plus or minus standard deviation (SD) together with *P*‐ and *Q*‐values. *P*‐values ≤0.05 were considered statistically significant if their corresponding *Q*‐values were ≤0.1. Student's *t*‐tests and Wilcoxon tests were performed in SAS Enterprise Guide 6.1 (SAS Institute, Cary, NC), and Benjamini and Hochberg corrections were performed in R 3.1.0 (https://www.r-project.org/).

Associations between plasma acylcarnitine levels and physiological measures were assessed from linear regression analyses. These analyses were performed on the pooled dataset of LBW and NBW individuals and were adjusted for age, BMI, and birth weight group. Only acylcarnitine levels that significantly differed between NBW and LBW individuals after the control or high‐fat, high‐calorie diet were included in the analyses. Data in Tables 4, 5, and 6 are presented as slope plus or minus SD together with *P*‐ and *Q*‐values. *P*‐values were considered statistically significant as described above. Linear regression analyses were performed in R.

## Results

Eighteen LBW and 25 NBW men were included in this study. Two LBW individuals of the recruited participants failed to consume all the food provided during the high‐fat, high‐calorie diet, and a NBW subject felt discomfort in connection with the clamp after the control diet and therefore did not further participate in this test in either the control or high‐fat, high‐calorie diet study part.

### Clinical characteristics

Low birth weight and NBW men displayed differences in body composition and glucose and lipid metabolism after the control and high‐fat, high‐calorie diets, and both birth weight groups showed changes in metabolism in response to the dietary challenge, as previously reported (Brons et al. 2008, 2012, 2013, 2015). A selection of variables that provide background for the current findings is shown in Tables 1 and 2 and also presented here.

**Table 1:** Clinical characteristics of low (LBW) and normal birth weight (NBW) men following the control (C) and high‐fat, high‐calorie (O) diets

	NBW (*n* = 25)			LBW (*n* = 18)			LBW versus NBW (*n* = 18, *n* = 25)		
	C (Mean ± SD)	O (Mean ± SD)	*P*_NBW_	C (Mean ± SD)	O (Mean ± SD)	*P*_LBW_	*P*_C_	*P*_O_	*P*_Δ_
**Anthropometry**									
Birth weight (g)	3901 ± 207	**–**	**–**	2717 ± 268	**–**	**–**	**≤0.001**	**–**	–
Weight (kg)	78.4 ± 9.3	78.6 ± 9.7	n.s.	77.1 ± 11.3	77.1 ± 11.4	n.s.	n.s.	n.s.	n.s.
Height (m)	1.83 ± 0.07	**–**	**–**	1.77 ± 0.05	**–**	**–**	**≤0.05**	**–**	–
Body mass index (kg/m^2^)	23.3 ± 2.4	23.3 ± 2.5	n.s.	24.6 ± 3.8	24.6 ± 3.8	n.s.	n.s.	n.s.	n.s.
**Lipid profiling**									
P‐TG (mmol/L)	0.92 ± 0.35	0.73 ± 0.35	**≤0.05**	1.07 ± 0.37	0.72 ± 0.24	**≤0.01**	n.s.	n.s.	n.s.
P‐CHOL (mmol/L)	4.36 ± 0.83	4.18 ± 0.82	n.s.	4.36 ± 0.78	4.27 ± 0.79	n.s.	n.s.	n.s.	n.s.
P‐VLDL‐CHOL (mmol/L)	0.42 ± 0.16	0.33 ± 0.16	**≤0.05**	0.49 ± 0.18	0.32 ± 0.12	**≤0.01**	n.s.	n.s.	n.s.
P‐LDL‐CHOL (mmol/L)	2.51 ± 0.72	2.28 ± 0.78	**≤0.05**	2.69 ± 0.76	2.57 ± 0.80	n.s.	n.s.	n.s.	n.s.
P‐HDL‐CHOL (mmol/L)	1.40 ± 0.22	1.56 ± 0.25	**≤0.01**	1.19 ± 0.23	1.38 ± 0.28	**≤0.01**	**≤0.01**	**≤0.05**	n.s.
**Clamp**									
* Basal*									
B‐Glucose (mmol/L)	4.59 ± 0.47	5.05 ± 0.40	**≤0.001**	4.97 ± 0.48	5.18 ± 0.34	**≤0.05**	**≤0.01**	n.s.	n.s.
S‐Insulin (pmol/L)	30.2 ± 14.7	43.4 ± 29.2	**≤0.05**	41.7 ± 14.6	44.7 ± 21.9	n.s.	**≤0.01**	n.s.	n.s.
S‐C‐peptide (pmol/L)	408 ± 146	529 ± 260	**≤0.01**	492 ± 116	539 ± 172	n.s.	**≤0.05**	n.s.	n.s.
P‐NEFA (μmol/L)	334 ± 136	205 ± 82	**≤0.001**	406 ± 200	188 ± 91	**≤0.001**	n.s.	n.s.	n.s.
HGP (mg/kg·FFM/min)	2.21 ± 0.48	2.85 ± 0.99	**≤0.01**	2.40 ± 0.5	2.48 ± 0.5	n.s.	n.s.	n.s.	**≤0.05**
Hepatic IR (mg/kg·FFM/min·pmol/L)	68.7 ± 34.1	113.7 ± 61.5	**≤0.001**	102.3 ± 50.8	108.7 ± 55.5	n.s.	**≤0.05**	n.s.	**≤0.05**
GOX (mg/kg·FFM/min)	2.34 ± 0.76	2.43 ± 0.71	n.s.	1.95 ± 0.78	2.20 ± 0.56	n.s.	n.s.	n.s.	n.s.
FOX (mg/kg·FFM/min)	1.00 ± 0.38	1.02 ± 0.33	n.s.	1.11 ± 0.53	1.17 ± 0.33	n.s.	n.s.	n.s.	n.s.
* Insulin‐stimulated*									
P‐NEFA (μmol/L)	9.29 ± 4.39	12.42 ± 6.43	**≤0.01**	9.56 ± 5.03	14.39 ± 7.76	**≤0.01**	n.s.	n.s.	n.s.
M‐value (mg/kg·FFM/min)	13.73 ± 2.32	13.29 ± 3.32	n.s.	13.47 ± 3.14	11.89 ± 3.57	**≤0.05**	n.s.	n.s.	n.s.
GOX (mg/kg·FFM/min)	5.18 ± 0.82	5.04 ± 0.98	n.s.	4.95 ± 0.92	4.78 ± 0.82	n.s.	n.s.	n.s.	n.s.
FOX (mg/kg·FFM/min)	0.01 ± 0.25	0.17 ± 0.33	n.s.	0.13 ± 0.46	0.37 ± 0.35	**≤0.05**	n.s.	**≤0.05**	n.s.
**IVGTT**									
FPIR (pmol/L)	1894 ± 1431	2604 ± 1793	**≤0.001**	2135 ± 1034	2750 ± 1509	**≤0.01**	n.s.	n.s.	n.s.
Hepatic DI	0.38 ± 0.63	0.25 ± 0.21	n.s.	0.21 ± 0.11	0.24 ± 0.13	n.s.	n.s.	n.s.	n.s.
Peripheral DI	0.29 ± 0.19	0.35 ± 0.20	**≤0.05**	0.33 ± 0.13	0.32 ± 0.17	n.s.	n.s.	n.s.	n.s.

Data are presented as mean ± SD. *P*‐values from Student's *t*‐tests are presented unadjusted for multiple comparisons, and *P*‐values ≤0.5 are considered statistically significant. *P*_NBW_ and *P*_LBW_: O versus C diet within each birth weight group, *P*_C_ and *P*_O_: LBW versus NBW individuals within each diet, *P*_Δ_: LBW versus NBW individuals on response values. n.s.: Not significant. *P*‐values ≤0.05 are marked in bold. Abbreviations: B, Blood; CHOL, Cholesterol; DI, Disposition index; FFM, Fat free mass; FOX, Fatty acid oxidation; FPIR, First‐phase insulin response; GOX, Glucose oxidation; HDL, High‐density lipoprotein; HGP, Hepatic glucose production; IR, Insulin resistance; IVGTT, Intravenous glucose tolerance test; LDL, Low‐density lipoprotein; NEFA, Nonesterified fatty acid; P, Plasma; S, Serum; TG, Triacylglycerol; VLDL, Very low‐density lipoprotein.

**Table 2:** Glucose, fatty acid, and protein oxidation rates and total energy expenditures in low (LBW) and normal birth weight (NBW) men during the control (C) and high‐fat, high‐calorie (O) diets

(kJ/min)	LBW versus NBW (*n* = 26)			LBW versus NBW (C: *n* = 20, O: *n* = 18)			LBW versus NBW (*n* = 20/*n* = 18, *n* = 26)		
	C (Mean ± SEM)	O (Mean ± SEM)	*P*_NBW_	C (Mean ± SEM)	O (Mean ± SEM)	*P*_LBW_	*P*_C_	*P*_O_	*P*_Δ_
**Calorimetry 24 h**
GOX									
Day	3.85 ± 0.17	3.50 ± 0.08	**0.0297**	3.69 ± 0.16	3.30 ± 0.14	0.0609	0.52	0.19	0.94
Night	1.97 ± 0.10	2.07 ± 0.07	0.3126	1.78 ± 0.09	1.84 ± 0.10	0.3391	0.18	0.06	0.97
Sleep	1.91 ± 0.12	1.89 ± 0.08	0.9131	1.58 ± 0.10	1.77 ± 0.11	0.0836	**0.05**	0.37	0.21
24 h	3.10 ± 0.13	2.93 ± 0.07	0.1510	2.92 ± 0.13	2.73 ± 0.09	0.2620	0.34	0.09	0.97
FOX									
Day	3.34 ± 0.16	4.23 ± 0.14	**<0.0001**	3.46 ± 0.14	4.52 ± 0.21	**<0.0001**	0.60	0.23	0.60
Night	2.34 ± 0.10	2.80 ± 0.10	**0.0005**	2.60 ± 0.08	3.06 ± 0.12	**0.0023**	0.07	0.10	0.93
Sleep	2.14 ± 0.14	2.72 ± 0.12	**0.0001**	2.50 ± 0.09	2.87 ± 0.13	**0.0221**	**0.05**	0.38	0.40
24 h	2.92 ± 0.12	3.63 ± 0.12	**<0.0001**	3.11 ± 0.11	3.91 ± 0.14	**<0.0001**	0.24	0.14	0.76
POX									
Day	1.13 ± 0.04	0.79 ± 0.03	**<0.0001**	1.08 ± 0.04	0.74 ± 0.04	**<0.0001**	0.48	0.32	0.71
Night	1.13 ± 0.04	0.79 ± 0.03	**<0.0001**	1.08 ± 0.04	0.74 ± 0.04	**<0.0001**	0.48	0.32	0.71
Sleep	1.13 ± 0.04	0.79 ± 0.03	**<0.0001**	1.08 ± 0.04	0.74 ± 0.04	**<0.0001**	0.48	0.32	0.71
24 h	1.13 ± 0.04	0.79 ± 0.03	**<0.0001**	1.08 ± 0.04	0.74 ± 0.04	**<0.0001**	0.48	0.32	0.71
EE									
Day	8.32 ± 0.15	8.52 ± 0.13	**0.0142**	8.24 ± 0.16	8.56 ± 0.18	**0.0021**	0.71	0.86	0.39
Night	5.43 ± 0.09	5.65 ± 0.10	**0.0001**	5.46 ± 0.11	5.66 ± 0.13	**0.0017**	0.82	0.97	0.99
Sleep	5.17 ± 0.09	5.39 ± 0.09	**0.0010**	5.16 ± 0.11	5.30 ± 0.13	**0.0009**	0.96	0.93	0.82
24 h	7.14 ± 0.12	7.36 ± 0.12	**0.0005**	7.12 ± 0.14	7.38 ± 0.15	**0.0008**	0.88	0.90	0.55

Data are presented as mean ± SEM. *P*‐values from Student's *t*‐tests are presented unadjusted for multiple comparisons, and *P*‐values ≤0.5 are considered statistically significant. *P*_NBW_ and *P*_LBW_: O versus C diet within each birth weight group, *P*_C_ and *P*_O_: LBW versus NBW individuals within each diet, *P*_Δ_: LBW versus NBW individuals on response values. *P*‐values ≤0.05 are marked in bold. *P*‐values on intervention effects are presented here for the first time. Details on the measurements have been described in previous articles (Brons et al. 2013, 2015). Abbreviations: EE, Energy expenditure; FOX, Fatty acid oxidation; GOX, Glucose oxidation; POX, Protein oxidation.

Low birth weight men had higher fasting blood glucose and serum insulin levels after the control diet compared with NBW men (Table 1). Also, LBW and NBW men both increased the fasting blood glucose level and decreased the fasting plasma nonesterified fatty acid level in response to overfeeding (Table 1). NBW men additionally increased the fasting serum insulin level due to this challenge. LBW and NBW men did not show differences in basal glucose or fatty acid oxidation rates after the control or high‐fat, high‐calorie diet when evaluated from the indirect calorimetry examination in connection with the clamp on the last study day, and they also did not change these rates in response to overfeeding (Table 1). However, when studied during the 24 h calorimetry during the interventions, LBW men had a higher fatty acid oxidation rate and a lower glucose oxidation rate during sleep on the control diet compared with NBW men (Table 2). Also, LBW and NBW men both increased fatty acid oxidation rates and decreased protein oxidation rates in all measured time intervals during the 24 h calorimetry in response to overfeeding, and NBW men decreased the glucose oxidation rate during day time due this challenge (Table 2). Furthermore, LBW and NBW men both increased total energy expenditures in response to overfeeding (Table 2). LBW men had a higher hepatic insulin resistance index after the control diet compared with NBW men, but LBW and NBW men did not have a different insulin‐stimulated glucose infusion rate, M‐value, after this diet (Table 1). Furthermore, NBW men increased the hepatic insulin resistance index in response to overfeeding, whereas LBW men decreased the M‐value in reaction to overfeeding. LBW and NBW men did not show a different first‐phase insulin response (FPIR) and nor different hepatic or peripheral disposition indices (DI) following the control or high‐fat, high‐calorie diet (Table 1). However, LBW and NBW men both increased the FPIR in response to overfeeding, and NBW men additionally increased the peripheral DI due to this challenge.

### Acylcarnitine levels and their relation to physiological measures

Low birth weight and NBW men showed differences in plasma acylcarnitine levels after the control diet, but not after the high‐fat, high‐calorie diet, and both birth weight groups furthermore showed changes in acylcarnitine levels in response to overfeeding (Table 3).

**Table 3:** Plasma acylcarnitine levels in low (LBW) and normal birth weight (NBW) men following the control (C) and high‐fat, high‐calorie (O) diets

(μmol/L)	NBW (*n* = 25)			LBW (*n* = 18)			LBW versus NBW (*n* = 18, *n* = 25)		
	C (Mean ± SD)	O (Mean ± SD)	*P*_NBW_ *Q*_NBW_	C (Mean ± SD)	O (Mean ± SD)	*P*_LBW_ *Q*_LBW_	*P*_C_ *Q*_C_	*P*_O_ *Q*_O_	*P*_Δ_ *Q*_Δ_
**Lipid profiling**									
Acylcarnitines									
C2	4.771 ± 0.797	3.985 ± 0.738	**0.0015**	5.985 ± 1.587	4.393 ± 0.784	**0.0007**	**0.0066**	0.0886	0.0595
			**0.0095**			**0.0046**	**0.0660**		
C3	0.317 ± 0.102	0.273 ± 0.075	**0.0487**	0.298 ± 0.103	0.259 ± 0.054	0.1582	0.5694	0.5058	0.8998
			0.1131						
C4/Ci4	0.146 ± 0.057	0.126 ± 0.037	0.1054	0.162 ± 0.047	0.123 ± 0.049	**0.0082**	0.3243	0.7823	0.2661
						**0.0291**			
C5:1	0.088 ± 0.040	0.073 ± 0.030	0.1275	0.084 ± 0.026	0.088 ± 0.039	0.5944	0.7246	0.1369	0.2252
C5's	0.122 ± 0.032	0.116 ± 0.030	0.5296	0.129 ± 0.029	0.128 ± 0.048	0.9683	0.4736	0.3526	0.7215
C4‐OH	0.038 ± 0.016	0.034 ± 0.010	0.3581	0.054 ± 0.019	0.030 ± 0.012	**<0.0001**	**0.0039**	0.2229	**0.0006**
						**0.0020**	**0.0520**		**0.0222**
C6	n.d.	n.d.	**–**	n.d.	n.d.	**–**	**–**	**–**	**–**
C5‐OH/C3‐DC	0.118 ± 0.047	n.d.	**–**	0.114 ± 0.054	n.d.	**–**	0.8051	**–**	**–**
C4‐DC/Ci4‐DC	0.033 ± 0.013	0.036 ± 0.017	0.5226	0.042 ± 0.013	0.044 ± 0.009	0.5642	**0.0328**	**0.0431**	0.9323
							0.1966	0.6667	
C8:1	0.100 ± 0.032	0.190 ± 0.054	**<0.0001**	0.116 ± 0.032	0.222 ± 0.067	**<0.0001**	0.1354	0.0957	0.3314
			**0.0013**			**0.0020**			
C8	0.122 ± 0.049	0.105 ± 0.030	0.1010	0.127 ± 0.042	0.111 ± 0.030	0.1193	0.6921	0.5241	0.9872
C5‐DC	0.048 ± 0.012	0.047 ± 0.015	0.7495	0.050 ± 0.013	0.050 ± 0.020	0.9169	0.7073	0.5795	0.7856
C8:1‐OH/C6:1‐DC	0.037 ± 0.009	0.062 ± 0.023	**<0.0001**	0.034 ± 0.014	0.060 ± 0.023	**0.0013**	0.3884	0.5660	0.5679
			**0.0013**			**0.0072**			
C6‐DC	0.064 ± 0.022	0.080 ± 0.022	**0.0039**	0.084 ± 0.017	0.089 ± 0.019	0.4862	**0.0032**	0.1926	0.1504
			**0.0165**				**0.0520**		
C10:3	n.d.	0.088 ± 0.031	**–**	0.053 ± 0.034	0.102 ± 0.035	**0.0005**	**–**	0.1965	**–**
						**0.0039**			
C10:2	n.d.	n.d.	**–**	n.d.	n.d.	**–**	**–**	**–**	**–**
C10:1	0.136 ± 0.053	0.121 ± 0.037	0.2216	0.138 ± 0.039	0.131 ± 0.023	0.5697	0.9120	0.3127	0.5908
C10	0.347 ± 0.160	0.311 ± 0.117	0.3218	0.297 ± 0.125	0.287 ± 0.102	0.8378	0.2736	0.4908	0.6401
C7‐DC	n.d.	n.d.	**–**	n.d.	n.d.	**–**	**–**	**–**	**–**
C8:1‐DC	0.024 ± 0.009	0.028 ± 0.012	0.1054	0.028 ± 0.012	0.026 ± 0.007	0.5224	0.1744	0.4497	0.1216
C10‐OH/C8‐DC	0.033 ± 0.014	0.032 ± 0.010	0.6502	0.048 ± 0.015	0.036 ± 0.010	**0.0048**	**0.0025**	0.1459	0.0585
						**0.0208**	**0.0520**		
C12:1	0.093 ± 0.040	0.067 ± 0.017	**0.0074**	0.109 ± 0.039	0.072 ± 0.016	**0.0005**	0.1226	0.3820	0.3802
			**0.0263**			**0.0039**			
C12	0.082 ± 0.037	0.083 ± 0.031	0.9887	0.089 ± 0.028	0.084 ± 0.024	0.5806	0.5344	0.8875	0.6768
C12‐OH/C10‐DC	0.008 ± 0.004	0.006 ± 0.003	**0.0327**	0.007 ± 0.002	0.006 ± 0.002	0.2281	0.5988	0.4714	0.3471
			**0.0866**						
C14:2	0.026 ± 0.015	0.018 ± 0.009	**0.0076**	0.031 ± 0.012	n.d.	**–**	0.3231	**–**	**–**
			**0.0263**						
C14:1	0.056 ± 0.018	0.049 ± 0.020	0.1900	0.071 ± 0.025	0.049 ± 0.016	**0.0054**	**0.0308**	0.8905	0.1259
						**0.0211**	0.1966		
C14	n.d.	n.d.	**–**	n.d.	0.026 ± 0.011	**–**	**–**	**–**	**–**
C14:1‐OH/C12:1‐DC	0.012 ± 0.006	0.011 ± 0.005	0.3108	0.014 ± 0.006	0.012 ± 0.005	0.2743	0.2065	0.2887	0.7372
C14‐OH/C12‐DC	0.008 ± 0.004	0.007 ± 0.003	0.1782	0.009 ± 0.002	0.008 ± 0.004	0.7148	0.4479	0.1429	0.5903
C16:2	0.006 ± 0.003	0.005 ± 0.003	**0.0199**	0.006 ± 0.002	0.004 ± 0.003	**0.0251**	0.9316	0.2900	0.3964
			**0.0630**			**0.0699**			
C16:1	0.019 ± 0.009	0.010 ± 0.005	**<0.0001**	0.020 ± 0.009	0.011 ± 0.007	**0.0121**	0.6717	0.5271	0.9987
			**0.0013**			**0.0393**			
C16	0.077 ± 0.012	0.066 ± 0.008	**0.0006**	0.083 ± 0.016	0.072 ± 0.015	**0.0133**	0.1681	0.1796	0.9050
			**0.0057**			**0.0399**			
C16:1‐OH/C14:1‐DC	0.008 ± 0.003	0.006 ± 0.003	**0.0022**	0.008 ± 0.003	0.006 ± 0.003	0.0912	0.7204	0.8956	0.8272
			**0.0119**						
C16‐OH/C14‐DC	0.005 ± 0.002	0.004 ± 0.002	0.2545	0.004 ± 0.002	0.005 ± 0.003	0.3113	0.2796	0.3719	0.1247
C18:2	0.032 ± 0.006	0.033 ± 0.008	0.6651	0.036 ± 0.013	0.036 ± 0.007	0.9486	0.3153	0.2563	0.8531
C18:1	0.096 ± 0.021	0.076 ± 0.016	**0.0012**	0.110 ± 0.024	0.083 ± 0.020	**0.0002**	**0.0488**	0.1802	0.4145
			**0.0091**			**0.0026**	0.2440		
C18	0.049 ± 0.008	0.042 ± 0.011	**0.0342**	0.051 ± 0.011	0.045 ± 0.012	**0.0307**	0.4460	0.3463	0.7884
			**0.0866**			**0.0798**			
C18:2‐OH	0.005 ± 0.004	n.d.	**–**	0.005 ± 0.005	0.004 ± 0.003	0.2252	0.7582	**–**	**–**
C18:1‐OH/C16:1‐DC	0.006 ± 0.002	0.004 ± 0.002	**0.0028**	0.007 ± 0.004	0.003 ± 0.002	**0.0015**	0.3136	0.3860	0.1537
			**0.0133**			**0.0073**			
C18‐OH/C16‐DC	0.006 ± 0.003	0.005 ± 0.003	0.6185	0.007 ± 0.003	0.006 ± 0.002	0.2424	**0.0344**	0.2525	0.4401
							0.1966		
C20:4	0.006 ± 0.003	0.006 ± 0.004	0.6264	0.007 ± 0.003	0.006 ± 0.004	0.5415	0.5908	0.8081	0.8447
C20	0.004 ± 0.002	0.005 ± 0.002	0.4190	0.006 ± 0.003	0.005 ± 0.003	0.3517	0.1378	0.6414	0.1991
C20:1‐OH/C18:1‐DC	0.008 ± 0.004	0.006 ± 0.002	0.0506	0.007 ± 0.003	0.007 ± 0.003	0.8446	0.6730	0.1213	0.1343
C20‐OH/C18‐DC	0.008 ± 0.003	0.009 ± 0.004	0.4546	0.009 ± 0.003	0.010 ± 0.002	0.6724	0.2669	0.5315	0.7204
C22	0.004 ± 0.002	0.005 ± 0.002	**0.0243**	0.005 ± 0.003	0.006 ± 0.002	0.2680	0.2850	0.5532	0.6343
			**0.0710**						
Total levels									
OH/DC	0.345 ± 0.061	0.377 ± 0.075	0.0511	0.412 ± 0.057	0.397 ± 0.041	0.3833	**0.0007**	0.2679	**0.0488**
Non‐OH/DC	6.674 ± 0.913	5.746 ± 0.865	**0.0006**	7.928 ± 1.822	6.215 ± 0.888	**0.0008**	**0.0049**	0.0901	0.0901
All	7.019 ± 0.945	6.122 ± 0.905	**0.0011**	8.340 ± 1.854	6.614 ± 0.885	**0.0009**	**0.0039**	0.0836	0.0790

Data are presented as mean ± SD, unless the acylcarnitine was not detected (n.d.) in more than 25% of the samples. *P*‐values ≤0.05 are presented together with *Q*‐values, and *P*‐values ≤0.05 with corresponding *Q*‐values ≤0.1 are considered statistically significant. *P*_NBW_ and *P*_LBW_, O versus C diet within each birth weight group; *P*_C_ and *P*_O_, LBW versus NBW individuals within each diet; *P*_Δ_, LBW versus NBW individuals on response values. *P*‐values ≤0.05 and *Q*‐values ≤0.1 are marked in bold.

Low birth weight men had higher C2, C4‐OH, C6‐DC, and C10‐OH/C8‐DC levels after the control diet compared with NBW men, and they also displayed higher total hydroxyl‐/dicarboxyl‐acylcarnitine and total acylcarnitine levels after this diet. However, LBW men did not have a higher total acylcarnitine level when C2 was excluded from the sum (data not shown). Furthermore, LBW and NBW men both decreased C2, C12:1, C16:2, C16:1, C16, C18:1, C18, C18:1‐OH/C16:1‐DC, and total acylcarnitine levels in response to overfeeding. LBW men additionally decreased C4/C4i, C4‐OH, C10‐OH/C8‐DC, and C14:1 levels when exposed to overfeeding, whereas NBW men decreased C12‐OH/C10‐DC, C14:2, and C16:1‐OH/C14:1‐DC levels due to this challenge. Moreover, LBW and NBW men both increased C8:1 and C8:1‐OH/C6:1‐DC levels in response to overfeeding. Also, LBW men increased the C10:3 level, whereas NBW men increased C6‐DC and C22 levels due to overfeeding.

C10‐OH/C8‐DC, total hydroxyl‐/dicarboxyl‐acylcarnitine, and total acylcarnitine levels were positively associated with the plasma nonesterified fatty acid level after the control diet (Tables 5 and 6). Also, C10‐OH/C8‐DC and total hydroxyl‐/dicarboxyl‐acylcarnitine levels tended to be negatively associated with the serum insulin level after the control diet. In addition, the C10‐OH/C8‐DC level tended to be positively associated with the M‐value after the control diet, and the total hydroxyl‐/dicarboxyl‐acylcarnitine level tended to be negatively associated with the hepatic insulin resistance index after this diet. Furthermore, the C4‐OH level was positively associated with the insulin‐stimulated glucose oxidation rate and negatively associated with the insulin‐stimulated fatty acid oxidation rate after the high‐fat, high‐calorie diet, and tended to be positively associated with the M‐value after this diet (Table 4). Moreover, a decrease in total hydroxyl‐/dicarboxyl‐acylcarnitine and total acylcarnitine levels in response to overfeeding was associated with a decrease in the plasma nonesterified fatty acid level, and a decrease in the total hydroxyl‐/dicarboxyl‐acylcarnitine level was additionally associated with a decrease in the plasma triacylglycerol level and an increase in FPIR.

**Table 4:** Associations between C2 or C4‐OH level and physiological measures following the control (C) and high‐fat, high‐calorie (O) diets and between response values (Δ)

	C2 (Slope, SD, *P*,* Q*)			C4‐OH (Slope, SD, *P*,* Q*)		
	C	O	Δ	C	O	Δ
**Lipid profiling**						
P‐TG	0.050	0.103	0.057	−1.540	−1.123	0.926
	0.039	0.054	0.045	2.817	3.623	3.132
	0.2140	0.0683	0.2079	0.5880	0.7580	0.7691
**Clamp**						
*Basal*						
B‐Glucose	−0.009	−0.023	−0.053	−0.881	−5.257	−0.954
	0.059	0.078	0.047	4.160	4.786	3.577
	0.8855	0.7649	0.2696	0.8335	0.2790	0.7912
S‐Insulin	−0.246	−2.109	−1.451	99.78	358.9	165.8
	1.389	2.893	1.368	96.49	178.0	100.4
	0.8604	0.4713	0.2970	0.3080	0.0522	0.1080
P‐NEFA	52.92	29.97	43.43	650.3	521.6	2038
	18.70	17.25	17.79	1335	1136	1274
	**0.0075**	0.0908	**0.0197**	0.6290	0.6490	0.1183
	0.1050		0.2352			
HGP	0.000	−0.078	−0.051	−0.222	−0.453	−1.593
	0.068	0.111	0.082	4.804	7.012	6.030
	0.9990	0.4875	0.5339	0.9630	0.9490	0.7931
Hepatic IR	1.222	−0.865	0.262	154.2	527.8	565.4
	4.012	10.31	5.149	283.6	653.4	387.0
	0.7625	0.9336	0.9600	0.5902	0.4247	0.1532
GOX	−0.115	−0.085	−0.129	−0.371	−4.375	−13.61
	0.111	0.159	0.099	7.973	10.82	7.284
	0.3070	0.5973	0.1990	0.9630	0.6883	0.0704
FOX	0.048	0.130	0.057	−0.678	−4.477	5.303
	0.065	0.068	0.051	4.604	4.455	3.854
	0.4640	0.0634	0.2790	0.8840	0.3219	0.1780
*Insulin‐stimulated*						
M‐value	0.306	0.974	0.016	2.371	69.74	−7.566
	0.286	0.547	0.289	21.08	34.32	21.17
	0.2920	0.0842	0.9560	0.9111	**0.0500**	0.7230
					0.1827	
GOX	−0.032	0.191	−0.166	−1.444	30.09	7.168
	0.122	0.196	0.128	9.430	10.84	9.759
	0.7930	0.3350	0.2040	0.8790	**0.0088**	0.4680
					**0.0616**	
FOX	0.017	−0.012	0.041	−2.894	−12.19	−7.563
	0.053	0.068	0.050	4.043	3.781	3.517
	0.7430	0.8619	0.4210	0.4790	**0.0027**	**0.0394**
					**0.0378**	0.2760
**IVGTT**						
FPIR	−100.5	−202.2	−181.9	4261	−8831	−1.0 E^4^
	113.5	309.3	94.15	8032	−2.1 E^4^	7553
	0.3819	0.5175	0.0618	0.5992	0.6700	0.1880
Hepatic DI	−0.012	−0.011	−0.029	−0.953	−1.588	−2.153
	0.016	0.032	0.013	1.062	2.107	1.044
	0.4430	0.7230	**0.0336**	0.3760	0.4560	**0.0470**
			0.2352			0.2760
Peripheral DI	−0.021	−0.031	−0.026	−1.774	0.2400	−2.457
	0.016	0.037	0.018	1.081	2.423	1.358
	0.1950	0.4080	0.1480	0.1104	0.9220	0.0795

Data are presented as slope, SD, and *P*‐value. *P*‐values ≤0.05 are presented together with *Q*‐values, and *P*‐values ≤0.05 with corresponding *Q*‐values ≤0.1 are considered statistically significant. *P*‐values ≤0.05 and *Q*‐values ≤0.1 are marked in bold. Regression analyses were performed on the pooled data set of LBW and NBW individuals and were adjusted for age, BMI, and birth weight group. Abbreviations: See Table 1.

**Table 5:** Associations between C6‐DC or C10‐OH/C8‐DC level and physiological measures following the control (C) and high‐fat, high‐calorie (O) diets and between response values (Δ)

	C6‐DC (Slope, SD, *P*,* Q*)			C10‐OH/C8‐DC (Slope, SD, *P*,* Q*)		
	C	O	Δ	C	O	Δ
**Lipid profiling**						
P‐TG	0.893	1.170	5.201	−1.976	0.774	5.816
	2.298	1.847	2.184	3.209	3.828	3.574
	0.6999	0.5302	**0.0227**	0.5420	0.8410	0.1124
			0.1589			
**Clamp**						
*Basal*						
B‐Glucose	−1.683	−5.724	−2.958	−7.441	−9.371	−4.710
	3.409	2.404	2.522	4.689	4.895	3.395
	0.6245	**0.0226**	0.2487	0.1211	0.0635	0.2392
		0.1848				
S‐Insulin	−113.9	−45.45	−16.15	−257.4	64.69	47.87
	77.68	96.33	70.67	102.1	225.3	114.1
	0.1512	0.6402	0.8210	**0.0162**	0.7760	0.6780
				0.1134		
P‐NEFA	2775	138.1	801.9	5505	894.5	3793
	992.8	602.5	978.8	1338	1169	1423
	**0.0082**	0.8200	0.4180	**0.0002**	0.4490	**0.0114**
	0.1148			**0.0028**		0.1596
HGP	0.662	3.164	1.891	−2.140	6.143	8.350
	3.900	3.712	4.235	5.419	7.286	6.613
	0.8660	0.3994	0.6578	0.6950	0.4050	0.2140
Hepatic IR	−296.3	−3561	−125.2	−397.3	−353.2	681.8
	224.6	336.6	268.9	315.9	760.8	416.6
	0.1956	0.2973	0.6440	0.2168	0.6454	0.1110
GOX	−11.80	−3.977	−10.08	−14.41	−0.277	−12.05
	6.210	5.189	5.197	8.846	10.78	8.477
	0.0654	0.4486	0.0608	0.1120	0.9796	0.1640
FOX	7.191	5.105	6.744	6.254	7.978	7.003
	3.567	2.203	2.627	5.190	4.642	4.322
	0.0513	**0.0264**	**0.0148**	0.2360	0.0945	0.1140
		0.1848	0.1589			
*Insulin‐stimulated*						
M‐value	33.49	20.55	5.711	46.84	23.39	1.585
	17.21	18.70	15.25	21.59	37.19	23.35
	0.0595	0.2793	0.7104	**0.0367**	0.5336	0.9463
				0.1713		
GOX	−1.617	5.470	1.158	−2.194	23.63	12.30
	7.505	6.240	6.853	11.41	12.31	11.49
	0.8310	0.3867	0.8670	0.8490	0.0630	0.2930
FOX	2.189	1.103	0.904	−1.085	−0.947	−1.110
	3.222	2.237	2.642	4.930	4.599	4.451
	0.5020	0.6249	0.7350	0.8270	0.8380	0.8047
**IVGTT**						
FPIR	−4457	−1.4 E^4^	−8198	−1.0 E^4^	−1.8 E^4^	−1.3 E^4^
	6603	1.0 E^4^	5370	9035	2.0 E^4^	8439
	0.5043	0.1780	0.1361	0.2615	0.3572	0.1193
Hepatic DI	−0.350	0.433	−0.131	0.483	−0.794	−0.566
	0.926	1.029	0.730	1.277	2.111	1.160
	0.7080	0.6770	0.8590	0.7080	0.7090	0.6290
Peripheral DI	0.2370	−0.119	0.267	0.192	−3.438	−1.464
	0.9630	1.193	1.024	1.329	2.437	1.585
	0.8070	0.9210	0.7960	0.8860	0.1672	0.3620

Data are presented as slope, SD, and *P*‐value. *P*‐values ≤0.05 are presented together with *Q*‐values, and *P*‐values ≤0.05 with corresponding *Q*‐values ≤0.1 are considered statistically significant. *P*‐values ≤0.05 and *Q*‐values ≤0.1 are marked in bold. Regression analyses were performed on the pooled data set of LBW and NBW individuals and were adjusted for age, BMI, and birth weight group. Abbreviations: See Table 1.

**Table 6:** Associations between total OH‐/DC‐acylcarnitine, total non‐OH‐/DC‐acylcarnitine, or total acylcarnitine level and physiological measures following the control (C) and high‐fat, high‐calorie (O) diets and between response values (Δ)

	OH/DC‐acylcarnitines (Slope, SD, *P*,* Q*)			Non‐OH/DC‐acylcarnitines (Slope, SD, *P*,* Q*)			All acylcarnitines (Slope, SD, *P*,* Q*)		
	C	O	Δ	C	O	Δ	C	O	Δ
**Lipid profiling**									
P‐TG	−0.210	0.714	2.119	0.045	0.101	0.071	0.042	0.099	0.074
	0.787	0.584	0.696	0.033	0.045	0.041	0.033	0.043	0.039
	0.7910	0.2294	**0.0043**	0.1888	**0.0308**	0.0893	0.2040	**0.0285**	0.0697
			**0.0315**		0.2793			0.2772	
**Clamp**									
*Basal*									
B‐Glucose	−1.605	−1.190	−0.246	−0.014	−0.665	−0.047	−0.016	−0.068	−0.045
	1.142	0.786	0.920	0.050	0.064	0.043	0.049	0.061	0.043
	0.1681	0.1387	0.7912	0.7778	0.3181	0.2898	0.7393	0.2781	0.2944
S‐Insulin	−54.46	−0.031	−5.130	−0.307	−1.698	−1.171	−0.389	−1.607	−1.131
	25.91	32.60	25.37	1.184	2.446	1.240	1.156	2.381	1.212
	**0.0427**	0.9920	0.8410	0.7970	0.4930	0.3520	0.7382	0.5050	0.3580
	0.1993								
P‐NEFA	1044	153.9	887.4	49.78	30.20	48.20	49.68	29.34	48.66
	339.9	188.6	292.7	15.62	14.16	15.81	15.18	13.74	15.34
	**0.0040**	0.4200	**0.0045**	**0.0029**	**0.0399**	**0.0043**	**0.0023**	**0.0396**	**0.0031**
	**0.0560**		**0.0315**	**0.0406**	0.2793	**0.0602**	**0.0322**	0.2772	**0.0434**
HGP	−0.402	0.212	0.191	0.009	−0.052	−0.042	0.008	−0.047	−0.039
	1.326	1.155	1.507	0.058	0.093	0.077	0.057	0.090	0.075
	0.7640	0.8551	0.8999	0.8710	0.5819	0.5910	0.8840	0.6040	0.6041
Hepatic IR	−160.9	−22.89	20.38	1.182	−0.209	1.137	0.835	−0.324	1.131
	73.23	114.1	95.36	3.400	8.697	4.678	3.323	8.461	4.568
	**0.0347**	0.8422	0.8320	0.7302	0.9810	0.8090	0.8030	0.9697	0.8060
	0.1993								
GOX	−2.652	0.924	−4.280	−0.125	−0.062	−0.147	−0.124	−0.053	−0.150
	2.156	1.759	2.032	0.094	0.136	0.091	0.092	0.131	0.089
	0.2270	0.6025	**0.0426**	0.1920	0.6514	0.1160	0.1840	0.6906	0.1020
			0.1491						
FOX	1.159	0.930	1.899	0.056	0.105	0.071	0.056	0.104	0.073
	1.256	0.772	1.017	0.055	0.057	0.048	0.053	0.055	0.047
	0.3620	0.2365	0.0704	0.3120	0.0736	0.1470	0.3040	0.0673	0.1310
*Insulin‐stimulated*									
M‐value	10.20	4.765	−2.950	0.332	0.799	0.012	0.334	0.776	0.004
	5.593	5.715	5.392	0.241	0.450	0.276	0.235	0.435	0.271
	0.0764	0.4102	0.5879	0.1770	0.0848	0.9669	0.1640	0.0832	0.9890
GOX	0.568	5.344	2.610	−0.030	0.245	−0.136	−0.028	0.259	−0.126
	2.590	1.917	2.603	0.105	0.160	0.123	0.102	0.153	0.121
	0.8280	**0.0085**	0.3240	0.7750	0.1340	0.2750	0.7870	0.0994	0.3050
		0.1190							
FOX	−0.509	−0.772	−1.380	0.019	−0.034	0.032	0.017	−0.036	0.028
	1.116	0.739	0.993	0.045	0.056	0.047	0.044	0.055	0.047
	0.6510	0.3035	0.1743	0.6810	0.5513	0.4987	0.7020	0.5124	0.5466
**IVGTT**									
FPIR	−1437	−4583	−4802	−1037	−232.8	−198.0	−101.4	−246.1	−201.0
	2214	3368	1810	95.87	260.0	85.64	93.56	252.7	83.68
	0.5206	0.1820	**0.0120**	0.2871	0.3765	**0.0270**	0.2862	0.3368	**0.0219**
			**0.0560**			0.1549			0.1533
Hepatic DI	−0.043	−0.136	−0.055	−0.011	−0.012	−0.027	−0.010	−0.013	−0.026
	0.308	0.347	0.269	0.013	0.027	0.012	0.013	0.026	0.012
	0.8890	0.6970	0.8380	0.4320	0.6470	**0.0332**	0.4390	0.6350	**0.0357**
						0.1549			0.1666
Peripheral DI	−0.185	−0.451	−0.329	−0.016	−0.030	−0.023	−0.016	−0.031	−0.023
	0.323	0.393	0.363	0.014	0.031	0.017	0.014	0.030	0.016
	0.5710	0.2590	0.3720	0.2490	0.3450	0.1700	0.2500	0.3150	0.1640

Data are presented as slope, SD, and *P*‐value. *P*‐values ≤0.05 are presented together with *Q*‐values, and *P*‐values ≤0.05 with corresponding *Q*‐values ≤0.1 are considered statistically significant. *P*‐values ≤0.05 and *Q*‐values ≤0.1 are marked in bold. Regression analyses were performed on the pooled dataset of LBW and NBW individuals and were adjusted for age, body mass index, and birth weight group. Abbreviations: See Table 1.

## Discussion

In order to investigate a possible differential and potentially incomplete fatty acid oxidation in LBW individuals, we measured fasting plasma levels of 45 acylcarnitine species or sets of species in 18 LBW and 25 NBW men following an isocaloric control diet and a 5‐day high‐fat, high‐calorie diet.

We demonstrated that LBW men had higher C2, C4‐OH, C6‐DC, and C10‐OH/C8‐DC levels after the control diet compared with NBW men, and also a higher total hydroxyl‐/dicarboxyl‐acylcarnitine level after this diet. Moreover, C10‐OH/C8‐DC and total hydroxyl‐/dicarboxyl‐acylcarnitine levels tended to be negatively associated with the serum insulin level after the control diet, and the total hydroxyl‐/dicarboxyl‐acylcarnitine level additionally tended to be negatively associated with the hepatic insulin resistance index after this diet.

**Figure 1:**
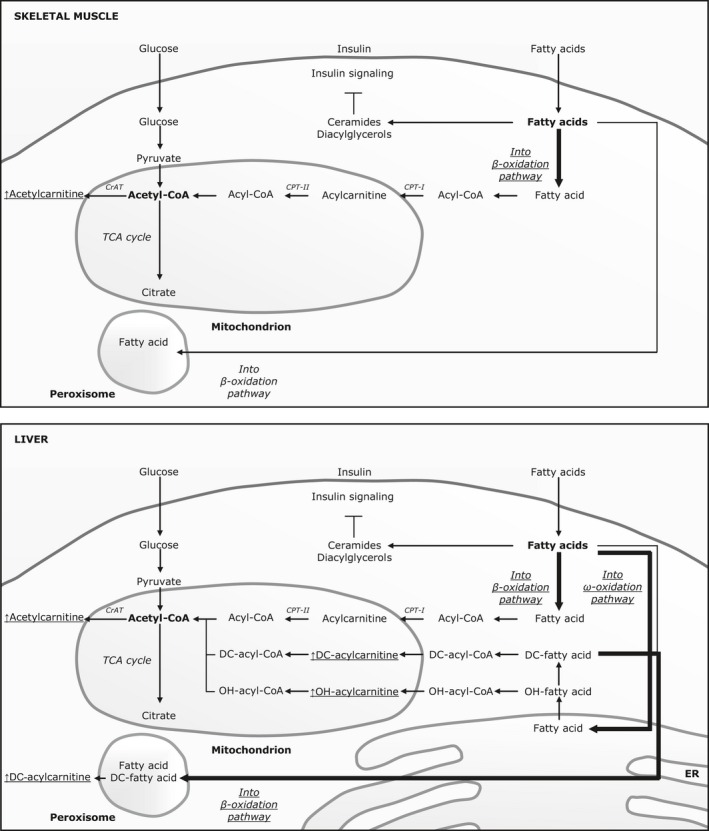
Proposed differences in fatty acid oxidation pathways between low (LBW) and normal birth weight (NBW) individuals. LBW individuals may have an increased beta‐oxidation in mitochondria of skeletal muscle (upper panel) and liver (bottom panel) that is not matched by an equivalently increased TCA cycle flux, resulting in an accumulation of acetyl‐CoA in the mitochondrial matrix and following of acetylcarnitine in the cytosol. Also, an excess of acetyl‐CoA could lead to an increased ketogenesis in the liver and following an accumulation of 3‐hydroxy‐butyrylcarnitine (not shown). Furthermore, LBW individuals may have an increased omega‐oxidation in the endoplasmic reticulum of the liver along with an increased beta‐oxidation of dicarboxyl‐fatty acids in peroxisomes, leading to an accumulation of hydroxyl‐ and dicarboxyl‐acylcarnitine species. Omega‐oxidation may be a scavenger pathway for oxidation of fatty acids that otherwise could be available for synthesis of lipotoxic lipid species such as ceramides and diacylglycerols that impair insulin signaling.

Low birth weight individuals’ higher C2 level after the control diet is reflective of an excess of acetyl‐CoA in the mitochondrial matrix. This indicates increased fatty acid, glucose, and/or amino acid oxidation rates relative to the TCA cycle flux (Fig. 1). Also, their unaltered total acylcarnitine level when excluding C2 suggests that they do not have limitations in fatty acid beta‐oxidation. In previous studies, we have found that LBW men have an increased fatty acid oxidation rate and a decreased glucose oxidation rate at night time during the control diet compared with NBW men, whereas they do not have a different protein oxidation rate (Brons et al. 2013). Thus, an accumulation of acetyl‐CoA in LBW men is likely to be due to an increased fatty acid beta‐oxidation. Furthermore, we have shown that LBW and NBW men do not show differences in expression levels of genes involved in oxidative phosphorylation or differences in ATP synthesis in skeletal muscle (Brons et al. 2008). Thus, at least in skeletal muscle, available data indicate that LBW individuals could have an increased beta‐oxidation rate and an unchanged TCA cycle flux, which would cause an accumulation of acetyl‐CoA in the mitochondrial matrix (Fig. 1). Their higher 3‐hydroxy‐butyrylcarnitine, C4‐OH, level is thought to reflect ketogenesis (McGarry and Foster 1972), consistent with an excess pool of acetyl‐CoA. As the liver is a primary site of ketogenesis, these findings may suggest increased rates of hepatic fatty acid oxidation in LBW men (Fig. 1). Hydroxyl‐ and dicarboxyl‐fatty acids are products of fatty acid omega‐oxidation in the endoplasmic reticulum of mainly the liver (Reddy and Hashimoto 2001), and with regards to medium‐chain dicarboxyl‐fatty acids and their acylcarnitine esters, in addition of beta‐oxidation in peroxisomes, as long‐chain dicarboxyl‐fatty acids derived from omega‐oxidation are oxidized in peroxisomes (Houten et al. 2012). Omega‐oxidation is a minor route for oxidation of fatty acids under normal physiological conditions (Reddy and Hashimoto 2001; Patsouris et al. 2006). However, the flux of fatty acids through this pathway is increased when intracellular levels of nonesterified fatty acids are high such as following high‐fat feeding (Patsouris et al. 2006) or under fasting (Patsouris et al. 2006) and starvation (Bjorkhem 1976; Kroetz et al. 1998). In addition to the omega‐oxidation pathway, hydroxyl‐fatty acids are intermediates in beta‐oxidation in mitochondria and peroxisomes (Reddy and Hashimoto 2001; Jones and Bennett 2010). Therefore, the higher C6‐DC, C10‐OH/C8‐DC, and total hydroxyl‐/dicarboxyl‐acylcarnitine levels in LBW men could reflect an increased omega‐oxidation (Mortensen and Gregersen 1981) along with an increased beta‐oxidation of dicarboxyl‐fatty acids (Houten et al. 2012) (Fig. 1), if these acylcarnitines are in fact both hydroxyl‐ and dicarboxyl‐ species, or, alternatively, an accumulation of intermediates in beta‐oxidation pathways, if the pooled species are comprised solely or predominantly of the hydroxyl‐species. Liquid chromatography‐tandem mass spectrometry (LC‐MS/MS) analyses will be required to resolve this issue, as opposed to the flow injection‐MS/MS analyses conducted herein. White adipose tissue lipolysis is increased during deficient adipose tissue insulin signaling, and we have previously described this trait in LBW individuals (Alibegovic et al. 2010). Furthermore, our recent studies of adipose tissue cells suggest that LBW is associated with an impaired development of subcutaneous adipose tissue (Ferland‐McCollough et al. 2012; Schultz et al. 2014). An increased lipolysis results in a shift in the equilibrium of fat storage from adipose tissue toward an increased storage in nonadipose tissue such as the liver (Samuel and Shulman 2016). Also, an increased hepatic fatty acid load is expected to induce omega‐oxidation (Patsouris et al. 2006). Interestingly, omega‐oxidation has been shown to be upregulated in experimental models of diabetes (Yoshioka et al. 1994; Miura 2013) as well as in patients with diabetes (Lippe et al. 1987). This, together with the present findings of a possible increase in omega‐oxidation in LBW individuals, suggests that an increased omega‐oxidation could be part of the metabolic phenotype of prediabetes and diabetes.

Acetylcarnitine, C2, has been reported to be a marker of prediabetes (Wang‐Sattler et al. 2012), and an elevated fasting plasma C2 level has been found in adults with type 2 diabetes (Adams et al. 2009; Villarreal‐Perez et al. 2014). Also, the fasting plasma C2 level has been shown to positively associate with the fasting plasma HbA1c level in women with or without diabetes (Adams et al. 2009). However, in this study, the C2 level did not associate with measures of insulin secretion or sensitivity. In addition, the fasting plasma C4‐OH level has been found to be elevated in obese women with type 2 diabetes (Fiehn et al. 2010). Furthermore, the fatty acid moiety of this acylcarnitine has been shown to interfere with insulin signaling (Tardif et al. 2001), and its levels in skeletal muscle have been associated with muscle insulin resistance in diet‐induced obesity rodent models (An et al. 2004). In this study, we did not observe any associations between the plasma C4‐OH level and insulin secretion or sensitivity. However, C10‐OH/C8‐DC and total hydroxyl‐/dicarboxyl‐acylcarnitine levels tended to be negatively associated with the serum insulin level, and the total hydroxyl‐/dicarboxyl‐acylcarnitine level additionally tended to be negatively associated with the hepatic insulin resistance index. This suggests that omega‐oxidation could act as a scavenger pathway for oxidation of fatty acids when intracellular acyl‐CoA levels are high to thereby reduce the availability of these precursors for the synthesis of lipotoxic lipid species such as ceramides and diacylglycerols that impair insulin signaling (Chavez and Summers 2012; Jornayvaz and Shulman 2012) (Fig. 1).

We furthermore demonstrated that LBW and NBW men decreased levels of 12 and 11 of the measured acylcarnitine species, respectively, as well as the total acylcarnitine level in response to overfeeding. In addition, they increased levels of three and four species, respectively, due to this challenge.

Low birth weight and NBW individuals’ decrease in several short‐, medium‐, and long‐chain acylcarnitine species, including C2, in response to overfeeding could be due to an increased fatty acid beta‐oxidation and TCA cycle flux following the high‐fat, high‐calorie diet compared to the control diet. This interpretation is strongly supported by the findings that LBW and NBW men increased both fatty acid oxidation rates and total energy expenditures during all time intervals of the 24 h calorimetry in response to overfeeding, and furthermore from the finding that they reduced the plasma nonesterified fatty acid level due to this challenge (Brons et al. 2012). In a prior rodent study, mice fed a high‐fat diet had higher serum levels of several medium‐ and long‐chain acylcarnitines compared with mice fed a standard diet (Koves et al. 2008). It was suggested that the high‐fat feeding resulted in an incomplete fatty acid beta‐oxidation (Koves et al. 2008). However, in this study, the high‐fat overfeeding was for only 5 days and the blood samples were collected after an overnight fast, as opposite to the rodent study in which the intervention was for 12 weeks, and the samples were collected in the fed state (Koves et al. 2008). Thus, an increased beta‐oxidation and TCA cycle flux in response to short‐term high‐fat overfeeding could be a compensatory mechanism to prevent an accumulation of lipids in nonadipose tissue. Such a mechanism is probably only transient and may not persist for long‐term high‐fat overfeeding exposure. This hypothesis, however, requires further studies. Their increase in C8:1 and C8:1‐OH/C6:1‐DC levels in response to overfeeding may be explained by the markedly higher n‐3 fatty acid content in the high‐fat, high‐calorie diet compared to the control diet (Table S1), as C8:1 and C6:1 n‐3 fatty acids are oxidation products of several n‐3 fatty acids, including alpha‐linolenic acid, C18:3 n‐3 fatty acid.

Our study is the first to describe fasting plasma acylcarnitine levels in LBW individuals with an increased risk of developing type 2 diabetes. It has its strengths in the careful selection of LBW and NBW men, highly standardized study setup, and in depth physiological and metabolic characterization of the individuals. In relation to the biological interpretation of the results, however, it has its limitations in the acylcarnitine profiling being on the plasma level, as plasma acylcarnitines represent the sum of contributions of acylcarnitines from various tissues, mainly skeletal muscle and liver, that may respond differently to a given metabolic challenge (Schooneman et al. 2013, 2014). Also, plasma and these tissues may have different turnover rates of acylcarnitines (Schooneman et al. 2014). In summary, we demonstrated that LBW men had higher C2 and C4‐OH levels after the control diet compared with NBW men, suggestive of an increased fatty acid beta‐oxidation in mitochondria relative to the TCA cycle flux. Furthermore, we showed that LBW men had higher C6‐DC, C10‐OH/C8‐DC, and total hydroxyl‐/dicarboxyl‐acylcarnitine levels, which may suggest an increased fatty acid omega‐oxidation in the endoplasmic reticulum of the liver concomitant with an increased beta‐oxidation in peroxisomes of omega‐oxidation‐derived dicarboxyl‐fatty acids. Interestingly, a cluster of short‐chain, dicarboxyl‐acylcarnitine species, including C6‐DC, has recently been shown to be prognostic for myocardial infarction and all‐cause cardiac mortality (Shah et al. 2010, 2012). Moreover, we found that C10‐OH/C8‐DC and total hydroxyl‐/dicarboxyl‐acylcarnitine levels tended to be negatively associated with the serum insulin level, and the total hydroxyl‐/dicarboxyl‐acylcarnitine level additionally tended to be negatively associated with the hepatic insulin resistance index. Therefore, we proposed that an increased fatty acid omega‐oxidation could prevent an accumulation of lipotoxic lipid species that impair insulin signaling in the liver. Intervention studies that aim to increase the efficiency of fatty acid oxidation, including hepatic omega‐oxidation, and TCA cycle flux to thereby potentially reduce an accumulation of lipids and improve insulin action in LBW individuals are needed.

## Conflicts of Interest

We all declare no financial or otherwise conflicts of interest in the study.

## Prior Publication

Preliminary results from this study has been published in abstract form in connection with The 51st European Association for the Study of Diabetes Annual Meeting, Stockholm, Sweden, 14–18 September 2015 (Ribel‐Madsen et al. 2015). The abstract can be found in the online version of Diabetologia and furthermore at: http://www.easdvirtualmeeting.org/users/31861.

## Supporting information




**Table S1.** Protein, carbohydrate, and fat contents of the control (C) and high‐fat, high‐calorie (O) diets.
**Table S2.** Acylcarnitine names, molecular formulas, methyl and butyl ester ion mass to charge ratios, and internal standards (IS) used for quantifications.
