# Supplementary material for: Plasma acylcarnitine profiling indicates increased fatty acid oxidation relative to tricarboxylic acid cycle capacity in young, healthy low birth weight men
Source: Physiol Rep. 2016 Sep 30;4(19):e12977. doi: 10.14814/phy2.12977 (PMC5064135; doi:10.14814/phy2.12977)
Supplement: Supplementary file 1 — Table S1. Protein, carbohydrate, and fat contents of the control (C) and high‐fat, high‐calorie (O) diets. Table S2. Acylcarnitine names, molecular formulas, methyl and butyl ester ion mass to charge ratios, and internal standards (IS) used for quantifications. [file PHY2-4-e12977-s001.docx]

**Supporting Information**

**Table S1**: **Protein, carbohydrate, and fat contents of the control (C) and high-fat, high-calorie (O) diets.**

|  | **C** | | **O** | | **O/C** | |
| --- | --- | --- | --- | --- | --- | --- |
|  | **Total**  (Mean) | **Per 100 g**  (Mean) | **Total**  (Mean) | **Per 100 g**  (Mean) | **Total**  (Ratio) | **Per 100 g**  (Ratio) |
| **Energy (kJ)** |  |  |  |  |  |  |
| Total | 9664 | 698 | 14848 | 1135 | 1.54 | 1.63 |
| **Energy (E%)** |  |  |  |  |  |  |
| Protein | 15 | 15 | 8 | 8 | 0.53 | 0.53 |
| Carbohydrate | 49 | 49 | 33 | 33 | 0.67 | 0.67 |
| Fat | 35 | 35 | 60 | 60 | 1.71 | 1.71 |
| **Energy (g)** |  |  |  |  |  |  |
| Protein | 88 | 6.4 | 67.1 | 5.1 | 0.76 | 0.80 |
| Carbohydrate | 266.7 | 19.2 | 277.6 | 21.2 | 1.04 | 1.10 |
| Fat | 92.1 | 6.6 | 239 | 18.3 | 2.60 | 2.77 |
| **Fat (g)** |  |  |  |  |  |  |
| Saturated fatty acids | 35.6 | 2.6 | 109.8 | 8.4 | 3.08 | 3.23 |
| Monounsaturated fatty acids | 31.5 | 2.3 | 85.4 | 6.5 | 2.71 | 2.83 |
| Polyunsaturated fatty acids | 8.6 | 0.6 | 28.5 | 2.2 | 3.31 | 3.67 |
| n-3 fatty acids | 0.9 | 0.1 | 5.8 | 0.4 | 6.44 | 4.00 |
| n-6 fatty acids | 7.2 | 0.5 | 21.8 | 1.7 | 3.03 | 3.40 |

**Table S2: Acylcarnitine names, molecular formulas, methyl and butyl ester ion mass to charge ratios, and internal standards (IS) used for quantifications.**

|  | **Names** | **Formulas** | **Methyl esters** | **Butyl esters** | **IS** |
| --- | --- | --- | --- | --- | --- |
|  |  |  | **[M+H]^+^** | **[M+H]^+^** |  |
| **Lipid profiling** |  |  |  |  |  |
| **Acylcarnitines** |  |  |  |  |  |
| C2 | Ethanoylcarnitine  (Acetylcarnitine) | C_9_H_17_NO_4_ | 218 | 260 | C2-IS |
| **C2-IS** | D_3_-Ethanoylcarnitine | C_9_D_3_H_14_NO_4_ | 221 | 263 | - |
| C3 | Propanoylcarnitine  (Propionylcarnitine) | C_10_H_19_NO_4_ | 232 | 274 | C3-IS |
| **C3-IS** | D_3_-Propanoylcarnitine | C_10_D_3_H_16_NO_4_ | 235 | 277 | - |
| C4/  Ci4 | Butanoylcarnitine  (Butyrylcarnitine)/  Isobutanoylcarnitine  (Isobutyrylcarnitine) | C_11_H_21_NO_4_ | 246 | 288 | C4-IS |
| **C4-IS** | D_3_-Butanoylcarnitine | C_11_D_3_H_18_NO_4_ | 249 | 291 | - |
| C5:1 | Pentenoylcarnitine  (Tiglylcarnitine) | C_12_H_21_NO_4_ | 258 | 300 | C5-IS |
| C5's | Pentanoylcarnitine  (Valerylcarnitine)/  Isopentanoylcarnitine  (Isovalerylcarnitine) | C_12_H_23_NO_4_ | 260 | 302 | C5-IS |
| C4-OH | 3-Hydroxybutanoylcarnitine | C_11_H_21_NO_5_ | 262 | 304 | C4-IS |
| **C5-IS** | D_9_-Isopentanoylcarnitine | C_12_D_3_H_20_NO_4_ | 269 | 311 | - |
| C6 | Hexanoylcarnitine  (Caproylcarnitine) | C_13_H_25_NO_4_ | 274 | 316 | C8-IS |
| C5-OH/  C3-DC | 3-Hydroxypentanoylcarnitine/  Propanedioylcarnitine  (Malonylcarnitine) | C_12_H_23_NO_5_/  C_10_H_17_NO_6_ | 276/  276 | 318/  360 | C8-IS |
| C4-DC/  Ci4-DC | Butanedioylcarnitine  (Succinylcarnitine)/  Isobutanedioylcarnitine | C_11_H_19_NO_6_ | 290 | 374 | C4-IS |
| C8:1 | Octenoylcarnitine | C_15_H_27_NO_4_ | 300 | 342 | C8-IS |
| C8 | Octanoylcarnitine  (Capryloylcarnitine) | C_15_H_29_NO_4_ | 302 | 344 | C8-IS |
| C5-DC | Pentanedioylcarnitine  (Glutarylcarnitine) | C_12_H_21_NO_6_ | 304 | 388 | C8-IS |
| **C8-IS** | D_3_-Octanoylcarnitine | C_15_D_3_H_26_NO_4_ | 305 | 347 | - |
| C8:1-OH/  C6:1-DC | 3-Hydroxyoctenoylcarnitine/  Hexenedioylcarnitine | C_15_H_27_NO_5_/  C_13_H_21_NO_6_ | 316/  316 | 358/  400 | C8-IS |
| C6-DC | Hexanedioylcarnitine  (Adipoylcarnitine) | C_13_H_23_NO_6_ | 318 | 402 | C8-IS |
| C10:3 | Decatrienoylcarnitine | C_17_H_27_NO_4_ | 324 | 366 | C8-IS |
| C10:2 | Decadienoylcarnitine | C_17_H_29_NO_4_ | 326 | 368 | C8-IS |
| C10:1 | Decenoylcarnitine | C_17_H_31_NO_4_ | 328 | 370 | C8-IS |
| C10 | Decanoylcarnitine  (Caprylcarnitine) | C_17_H_33_NO_4_ | 330 | 372 | C8-IS |
| C7-DC | Heptanedioylcarnitine  (Pimeloylcarnitine) | C_14_H_25_NO_6_ | 332 | 416 | C8-IS |
| C8:1-DC | Octenedioylcarnitine | C_15_H_25_NO_6_ | 344 | 428 | C8-IS |
| C10-OH/  C8-DC | 3-Hydroxydecanoylcarnitine/  Octanedioylcarnitine  (Suberoylcarnitine) | C_17_H_33_NO_5_/  C_15_H_27_NO_6_ | 346/  346 | 388/  430 | C8-IS |
| C12:1 | Dodecenoylcarnitine  (Lauroleoylcarnitine) | C_19_H_35_NO_4_ | 356 | 398 | C8-IS |
| C12 | Dodecanoylcarnitine  (Lauroylcarnitine) | C_19_H_37_NO_4_ | 358 | 400 | C8-IS |
| C12-OH/  C10-DC | 3-Hydroxydodecanoylcarnitine/  Decanedioylcarnitine  (Sebacoylcarnitine) | C_19_H_37_NO_5_/  C_17_H_31_NO_6_ | 374/  374 | 416/  458 | C16-IS |
| C14:2 | Tetradecadienoylcarnitine | C_21_H_37_NO_4_ | 382 | 424 | C16-IS |
| C14:1 | Tetradecenoylcarnitine  (Myristoleoylcarnitine) | C_21_H_39_NO_4_ | 384 | 426 | C16-IS |
| C14 | Tetradecanoylcarnitine  (Myristoylcarnitine) | C_21_H_41_NO_4_ | 386 | 428 | C16-IS |
| C14:1-OH/  C12:1-DC | 3-Hydroxytetradecenoylcarnitine/  Dodecenedioylcarnitine | C_21_H_39_NO_5_/  C_19_H_33_NO_6_ | 400/  400 | 442/  484 | C16-IS |
| C14-OH/  C12-DC | 3-Hydroxytetradecanoylcarnitine/  Dodecanedioylcarnitine | C_21_H_41_NO_5_/  C_19_H_35_NO_6_ | 402/  402 | 444/  486 | C16-IS |
| C16:2 | Hexadecadienoylcarnitine  (Palmitolinoleoylcarnitine) | C_23_H_41_NO_4_ | 410 | 452 | C16-IS |
| C16:1 | Hexadecenoylcarnitine  (Palmitoleoylcarnitine) | C_23_H_43_NO_4_ | 412 | 454 | C16-IS |
| C16 | Hexadecanoylcarnitine  (Palmitoylcarnitine) | C_23_H_45_NO_4_ | 414 | 456 | C16-IS |
| **C16-IS** | D_3_-Hexadecanoylcarnitine | C_23_D_3_H_42_NO_4_ | 417 | 459 | - |
| C16:1-OH/  C14:1-DC | 3-Hydroxyhexadecenoylcarnitine/  Tetradecenedioylcarnitine | C_23_H_43_NO_5_/  C_21_H_37_NO_6_ | 428/  428 | 470/  512 | C16-IS |
| C16-OH/  C14-DC | 3-Hydroxyhexadecanoylcarnitine/  Tetradecanedioylcarnitine | C_23_H_45_NO_5_/  C_21_H_39_NO_6_ | 430/  430 | 472/  514 | C16-IS |
| C18:2 | Octadecadienoylcarnitine  (Linoleoylcarnitine) | C_25_H_45_NO_4_ | 438 | 480 | C16-IS |
| C18:1 | Octadecenoylcarnitine  (Oleylcarnitine) | C_25_H_47_NO_4_ | 440 | 482 | C16-IS |
| C18 | Octadecanoylcarnitine  (Stearoylcarnitine) | C_25_H_49_NO_4_ | 442 | 484 | C16-IS |
| C18:2-OH | 3-Hydroxyoctadecadienoylcarnitine | C_25_H_45_NO_5_ | 454 | 496 | C16-IS |
| C18:1-OH/  C16:1-DC | 3-Hydroxyoctadecenoylcarnitine/  Hexadecenedioylcarnitine | C_25_H_47_NO_5_/  C_23_H_41_NO_6_ | 456/  456 | 498/  540 | C16-IS |
| C18-OH/  C16-DC | 3-Hydroxyoctadecanoylcarnitine/  Hexadecanedioylcarnitine | C_25_H_49_NO_5_/  C_23_H_43_NO_6_ | 458/  458 | 500/  542 | C16-IS |
| C20:4 | Eicosatetraenoylcarnitine  (Arachidonoylcarnitine) | C_27_H_45_NO_4_ | 462 | 504 | C16-IS |
| C20 | Eicosanoylcarnitine  (Arachidoylcarnitine) | C_27_H_53_NO_4_ | 470 | 512 | C16-IS |
| C20:1-OH/  C18:1-DC | 3-Hydroxyeicosenoylcarnitine/  Octadecenedioylcarnitine | C_27_H_51_NO_5_/  C_25_H_45_NO_6_ | 484/  484 | 526/  568 | C16-IS |
| C20-OH/  C18-DC | 3-Hydroxyeicosanoylcarnitine/  Octadecanedioylcarnitine | C_27_H_53_NO_5_/  C_25_H_47_NO_6_ | 486/  486 | 528/  570 | C16-IS |
| C22 | Docosanoylcarnitine  (Behenoylcarnitine) | C_29_H_57_NO_4_ | 498 | 540 | C16-IS |
